# Molecular characteristics of representatives of the genus *Brachylecithum* Shtrom, 1940 (Digenea, Dicrocoeliidae) with comments on life cycle and host specificity

**DOI:** 10.1007/s00436-015-4875-3

**Published:** 2015-12-28

**Authors:** Joanna Hildebrand, Jilji Sitko, Grzegorz Zaleśny, Witold Jeżewski, Zdzisław Laskowski

**Affiliations:** Department of Parasitology, Institute of Genetics and Microbiology, University of Wrocław, Przybyszewskiego 63, 51-148 Wrocław, Poland; Comenius Museum, Horní nám. 7, 750 11 Přerov, Czech Republic; Department of Invertebrates Systematic and Ecology, Institute of Biology, Wrocław University of Environmental and Life Sciences, Kożuchowska 5b, 51-631 Wrocław, Poland; Institute of Parasitology, Polish Academy of Sciences, Twarda 51/55, 00-818 Warszawa, Poland

**Keywords:** Digenea, Dicrocoeliidae, *Brachylecithum*, Molecular phylogeny, Life cycle abbreviation

## Abstract

The genus *Brachylecithum* was for the first time subject to molecular taxonomic phylogenetic analysis in order to ascertain relationships among its component taxa. We used two markers—the nuclear ribosomal 28S ribosomal DNA (rDNA) gene and the mitochondrial *cox*1 gene, for six species of the genus; 11 sequences of partial 28S rDNA and partial *cox*1 were obtained from adult *B. capilliformis*, *B. glareoli*, *B. kakea*, *B. laniicola*, *B. lobatum*, and *B. strigis*, and from larval stages obtained from snails of the genus *Cepaea*. We propose to synonymize *B. strigis* with *B. lobatum*, while the genetic differences in the 28S rDNA gene and mitochondrial *cox*1 gene confirm the species status of *B. capilliformis* and indicate a distinct group within *Brachylecithum*, including *B. kakea* and *B. laniicola*. Cercarial and metacercarial isolates from the snails showed 100 % similarity to *B. lobatum*; thus, it is the first record of *Cepaea* snails as intermediate hosts of this species and the first report on life cycle abbreviation within the Dicrocoeliidae.

## Introduction

Species of the family Dicrocoeliidae Looss, 1899 complete their life cycle on land. Adults mainly parasitize the bile ducts and gall bladders of birds and mammals. The family is speciose and shows a high level of morphological variability or phenotypic plasticity. In her review of the Dicrocoeliidae, Pojmańska (2008) stated that the great variation of body shape, size, and topography of the internal organs made it difficult to propose an unambiguous classification system; consequently, different authors proposed several taxonomic arrangements. The family’s classification was modified on several occasions; new genera, subfamilies, or tribes were proposed, and species were transferred between genera by, for example, Shtrom, Travassos, Skrjabin and Evranova, Yamaguti, Odening, or Panin (Pojmańska 2008). The most recent classification of the group is mainly based on adult morphological characters, and knowledge of the biology and life cycles of most species is scanty.

Molecular data on dicrocoeliid genera are incomplete, and phylogenetic analyses among the numerous dicrocoeliid taxa are unclear and often limited to a single species (e.g., Maurelli et al. 2007; Cai et al. 2012). The GenBank contains such data for 17 species (except those of *Dicrocoelium* and *Eurytrema* species) for which only sequences of partial 18S and 28S ribosomal DNA (rDNA) genes are available. The Dicrocoeliidae comprise over 400 species (Pojmańska 2008), and the scarcity of molecular information limits phylogenetic analysis within the family. So far, only publications of Tkach et al. (2001) and Olson et al. (2003) outlined the position of this family among the digeneans, and two studies dealt with individual species in a phylogenetic context: Ribas et al. (2012) on *Paraconcinnum leirsi* from African rodents and Hildebrand et al. (2015) on *Lyperosomum sarotruhe* from African birds.

More than 90 representatives of *Brachylecithum* Shtrom, 1940 were described in Panin’s (1984) publication, which is the most recent monograph on the Dicrocoeliidae. However, some of the descriptions were based on single specimens from one locality, and the host specificity was a common criterion in describing new species. The genus *Brachylecithum*, like *Brachydistomum* Travassos, 1944, *Corrigia* Shtrom, 1940, and *Lutztrema* Travassos 1941, represents the subfamily Dicrocoeliinae, whose life cycles include long-tailed xiphidocercariae which leave their first intermediate host, the land snail, in mucoid balls. Arthropods are the second intermediate hosts. Most experimental studies on the life cycles of *Brachylecithum* concerned *B. alfortense* (Railliet, 1900), according to Rysavy (1960) a synonym of *B. lobatum*; *B. americanum* Denton, 1945; *B. mosquensis* (Skrjabin and Isaitschikoff, 1927); *B. myadestis* Carney, 1972; *B. orfi* Kingston and Freeman, 1959 and *B. stunkardi* (Pande, 1939) (Carney 1970, 1972, 1974; Denton 1945; Kingston 1965; Timon-David 1957). It should be emphasized that knowledge of the second intermediate hosts of *Brachylecithum* is still incomplete. Only Denton (1945) suggested that chrysomelid beetles served as the natural second intermediate hosts of *B. americanum. Brachylecithum* is one of the largest dicrocoeliine genera whose representatives are found mostly in birds; however, the systematic position of some species, their phylogenetic relationships, and host associations remain unclear.

Our studies yielded new sequence data for the nuclear and mitochondrial markers of ten representatives of *Brachylecithum* (from various hosts and different development stages). The material came from central Europe. To our knowledge, this study is the first attempt at phylogenetic analysis of relationships within the genus.

## Material and methods

### Sample collection

**Fig. 1:**
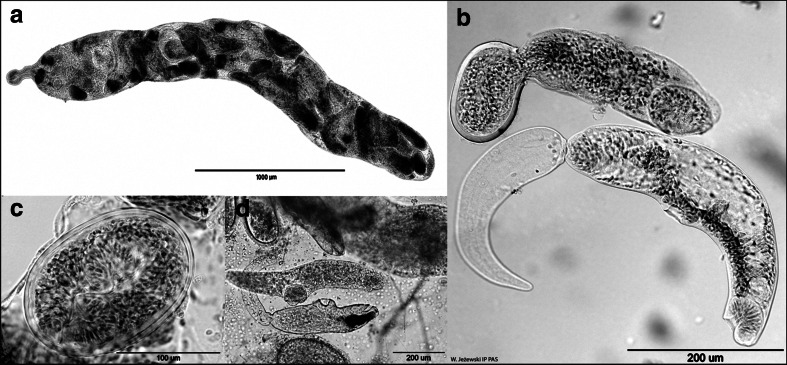
Larval stages of *Brachylecithum lobatum* from *Cepaea hortensis*. **a** Sporocyst, **b** cercaria and metacercaria hatching from the cyst, **c** encysted metacercaria, and **d** cercaria, free metacercaria, cysts with metacercaria, and fragment of a sporocyst

Adult specimens of *Brachylecithum* Shtrom, 1940 used in this study were collected and identified during long-term helminthological studies of birds from the Czech Moravia, with some additional birds sampled in Poland (Mazovia Disctrict, Baltic Coast) within parasitological research of the Institute of Parasitology, PAS, and the University of Wrocław. Adult *B. glareoli* were collected from the bank vole *Myodes glareolus* live-trapped in the water intake area of Wrocław (Lower Silesia, Poland). Larval stages of *Brachylecithum* spp. were isolated from land snails *Cepaea* spp. (Fig. 1) in the Mazovia District during the research on the helminth fauna of slugs and their role in the spread of parasites in natural habitats. In some of the examined specimens of the white-lipped (*Cepea hortensis*) and brown-lipped snail (*C. nemoralis*), we found sporocysts with cercariae, while in others the sporocysts contained encysted metacercariae. The larval stages were collected alive, washed in physiological salt solution, heat-killed in tap water, and preserved in 70 % ethanol. Prior to fixation, measurements and photographs were taken with an Olympus DP25 digital camera coupled with an Olympus BX50 light microscope. The adults were identified under the microscope, rinsed in saline and water, and fixed in 70 or 96 % ethanol for further morphological or molecular processing. The names of the dicrocoeliids, their hosts, and localities are listed in Table 1.

**Table 1:** The list of taxa used in this study, host species, geographical origin of material, and GenBank accession numbers

Digenea taxa	Host species	Geographical origin	GenBank accession no.	
			28S	*cox*1
*Brachylecithum capilliformis* Oshmarin, 1952	River warbler	Czech Republic, Central Moravia	KU212184^a^	KU212182^a^
	*Locustella fluviatilis*			
*Brachylecithum glareoli* Hildebrand, Okulewicz and Popiołek, 2007	Bank vole	Poland, Lower Silesia	KU212201^a^	KU212202^a^
	*Myodes glareolus*		KU212203^a^	KU212204^a^
*Brachylecitum grummti* Odening, 1964	Cinnamon attila	Brazil	KP765768	–
	*Attila cinnamomeus*			
*Brachycecithum kakea* (Bhalerao, 1926)	Great reed warbler	Czech Republic, Central Moravia	KU212178^a^	KU212197^a^
	*Acrocephalus arundinaceus*		KU212180^a^	KU212181^a^
*Brachylecithum lanicola* (Layman, 1926)	Red-backed shrike	Czech Republic, Central Moravia	KU212183^a^	KU212194^a^
	*Lanius collurio*			
*Brachylecithum lobatum* (Railliet, 1900)	the carrion crow	Czech Republic, Central Moravia	AY222260	–
	*Corvus cornae*			
*Brachylecithum lobatum* (Railliet, 1900)	Rook	Czech Republic, Central Moravia	KU212200^a^	KU212199^a^
	*Corvus frugilegus*			
*Brachylecithum lobatum* (Railliet, 1900)	Eurasian sparrowhawk	Poland, Baltic coast	KU212179^a^	KU212198^a^
	*Accipiter nisus*			
*Brachylecithum lobatum* (Railliet, 1900) sporocysts with cercariae	White-lipped snail	Poland, Mazovia	KU212189^a^	KU212190^a^
	*Cepaea hortensis*			
*Brachylecithum lobatum* (Railliet, 1900) metacercaria	White-lipped snail	Poland, Mazovia	KU212187^a^	KU212186^a^
	*Cepaea hortensis*			
*Brachylecithum lobatum* (Railliet, 1900) sporocysts with cercariae	Brown-lipped snail	Poland, Mazovia	KU212188^a^	KU212185^a^
	*Cepaea nemoralis*			
*Brachylecithum strigis* (Yamaguti, 1939)	European scops owl	Czech Republic, Central Moravia	KU212195^a^	KU212196^a^
	*Otus scops*			
*Dicrocoelium dendriticum* (Rudolphi, 1819)	Bobak marmot	Ukraine	AF151939	–
	*Marmota bobak*			
*Lyperosomum collurionis* (Skrjabin and Isaichikov, 1927)	Eurasian blackcap	Czech Republic, Central Moravia	AY222259	–
	*Sylvia atricapilla*			
*Lyperosomum collurionis* (Skrjabin and Isaichikov, 1927)	Eurasian blackcap	Czech Republic, Central Moravia	KU212193^a^	KU212192^a^
	*Sylvia atricapilla*			
*Lyperosomum transcarpathicus* Bychowskaja-Pavlovskaja and Kulakova, 1978	Eurasian pygmy shrew	Ukraine	AF151943	–
	*Sorex minutus*			
*Macvicaria magellanica* Laskowski, Jeżewski and Zdzitowiecki, 2013	Nototheniid fish	Antarctica	KU212191^a^	–
	*Patagonotothen* spp.			

^a^New sequences generated by this study

### DNA extraction, gene amplification, and sequencing

For molecular analysis, DNA was extracted from ethanol-fixed, single specimens of adults, metacercariae, and sporocysts (with cercariae) using the Qiagen DNeasy™ tissue kit and Genoplast Tissue Genomic DNA Extraction Mini Kit according to the manufacturer’s instructions. The partial nuclear ribosomal 28S rDNA (D1–D3) gene was amplified using the forward primer DLS1 and the reverse primer 1500R. The thermocycling profile was as follows: 3 min denaturation at 94 °C; 35 cycles of 30 s at 94 °C, 1 min at 55 °C, and 1 min at 72 °C; and 10 min extension at 72 °C. The partial mitochondrial cytochrome *c* oxidase subunit 1 (*cox*1) gene was amplified using the forward primers COIA3/COIDF1 and the reverse primers COITR1/COIDR1 with the following thermocycling profile: 3 min denaturation at 94 °C; 35 cycles of 30 s at 94 °C, 30 s at 50 °C, and 1 min at 72 °C; and 5 min extension at 72 °C. PCR and sequencing primers are listed in Table 2. The amplified products were purified using a PCR purification kit (Genoplast, Poland) according to the manufacturer’s instruction and sequenced directly on an ABI Prism 373xl automated sequencer (Applied Biosystems, USA) using ABI BigDye™ (Applied Biosystems) by Genomed (Poland). DNA products were sequenced in both directions using the PCR primers and sequencing primers (lsrDNA). Forward and reverse sequences were assembled and aligned using Vector NTI Advance 11.0 (Invitrogen, Life Technologies) software and submitted to GenBank. Accession numbers of adults and larvae are listed in Table 2.

**Table 2:** Sequences of primers used in the analyses

Name	Sequence (5′–3′)	Reference
*cox*1 PCR and sequencing primers		
COIA3	GTTGCATGATACTTGGTTTGTTG	Present study
COITR1	CAACAACAAACCAAGTATCATG	Laskowski and Rocka 2014
COIDF1	TATTGTTTCAGCATATGTTTTG	Present study
COIDR1	CAACAAACCAAGTATCATGCAAC	Present study
28S PCR primers		
DLS1	ACCCGCTGAACTTAAGCATATCACTAAGC	Laskowski and Rocka 2014
1500R	GCTATCCTGAGGGAAACTTCG	Tkach et al. 2003
28S sequencing primers		
DF400	AAACCGCTCAGAGGTAAGC	Present study
1100R	CTTGGTCCGTGTTTCAAGACGGG	Present study

### Sequence alignment and phylogenetic analyses

The first phylogenetic analysis was based on partial 28S rDNA gene with the newly generated sequences of *Brachylecithum* and selected sequences of dicroceoliids from GenBank (Table 1). The nucleotides were aligned with AlignX (Vector NTI Advance 11.0), with default settings. Regions that could not be unambiguously aligned were excluded from the analysis. JModelTest version 2.1.4 (Darriba et al. 2012; Guindon and Gascuel 2003) was used to select models of evolution using the Akaike information criterion (AIC). The chosen parameters of the substitution model were GTR + G. Phylogenetic trees were constructed using Bayesian inference (BI) as implemented in the MrBayes program version 3.2 (Ronquist and Huelsenbeck 2003) with *Macvicaria magellanica* (Opecoelidae) (Laskowski et. al. 2013) as an outgroup.

The sequences of a partial region of the cytochrome *c* oxidase subunit 1 gene were obtained for adults of *Brachylecithum* and *Lyperosomum* as well as from sporocysts, cercariae, and metacercariae isolated from their snail hosts (Table 1). All generated mitochondrial DNA sequences were used in the next analysis using the same parameters and the same software as in the 28S rDNA analysis, with the HKY + G + I models of evolution.

Subsequently, partial 28S rDNA sequences were concatenated with partial mitochondrial *cox*1 sequences in the second dataset for independent phylogenetic analysis. In mixed analyses (28S + *cox*1), the data were partitioned into two character sets: (1) 28S and (2) mt data as nucleotides. The phylogenetic analysis was carried out using the BI in the MrBayes program. Likelihood settings were set to the GTR + G model (28S) and HKY + G (*cox*1) in accordance with the AIC results. In concatenated datasets, parameters were estimated separately. The analysis of combined sequences of 28S + *cox*1 was provided with *Lyperosomum collurionis* as an outgroup (based on 28S rDNA analysis). Phylogenetic trees were visualized using the TreeView software (Page 1996). The evolutionary divergence between the sequences was estimated using MEGA6 software (Tamura et al. 2013); analyses of the number of nucleotide substitutions were conducted using the maximum composite likelihood model (Tamura et al. 2004).

## Results

Eleven partial 28S rDNA sequences (1198 bp) and partial *cox*1 sequences (388 bp) were obtained from adult trematodes of the genus *Brachylecithum*: *B. capilliformis*, *B. glareoli*, *B. kakea*, *B. laniicola*, *B. lobatum*, and *B. strigis*, and from larvae obtained from snails. Additionally, we obtained sequences of *cox*1 for adult *L. collurionis*.

The ribosomal sequences of adults of *B. lobatum* from *Corvus frugilegus* and *Accipiter nisus*, *B. strigis* from *Otus scops*, and all the larval forms (sporocysts, cercariae, and metacercariae) from *Cepaea* spp. were identical with the sequence of *B. lobatum* from *Corvus cornae* previously deposited in the GenBank (AY222260) and with *B. glareoli* from *M. glareolus*.

**Fig. 2:**
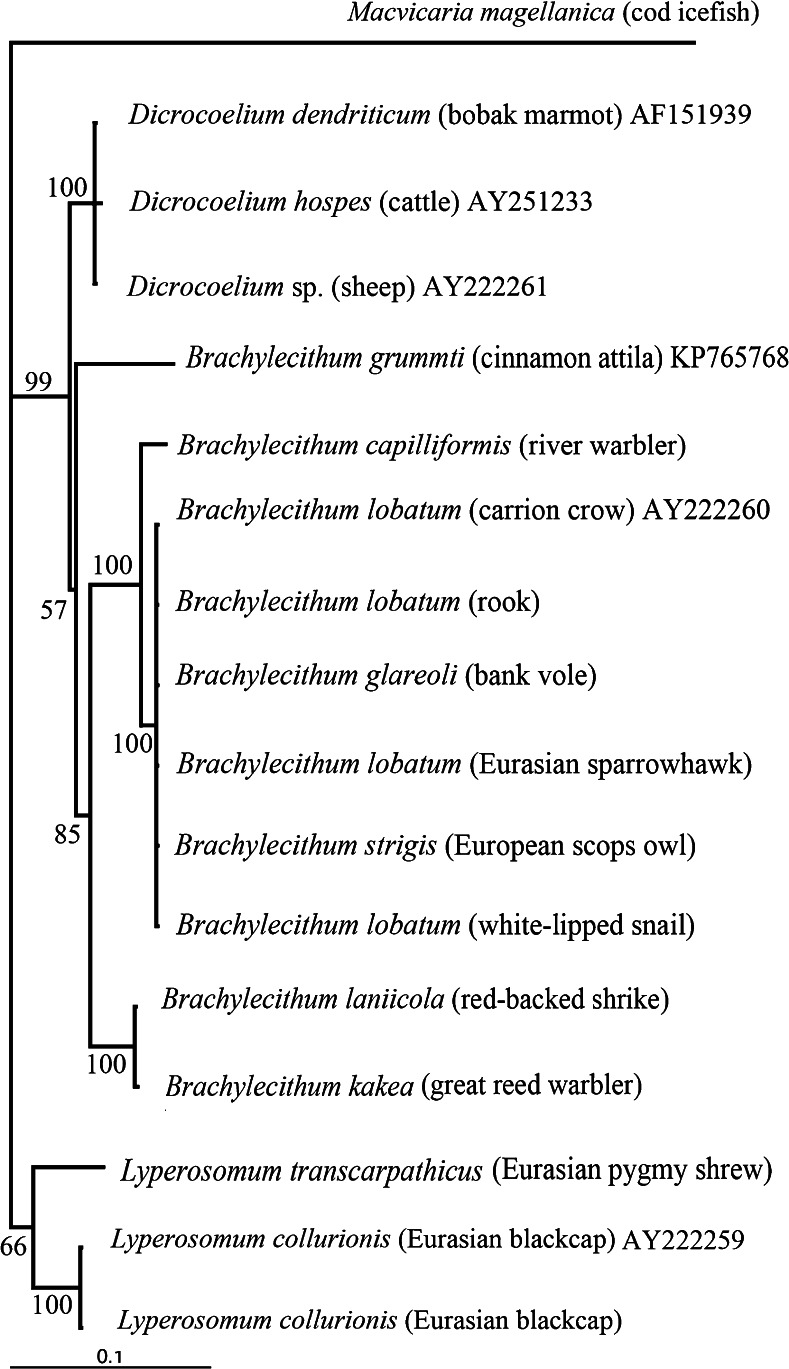
Bayesian analysis of partial sequences of the 28S rDNA gene of 16 members of Dicrocoeliidae. The tree constructed with MrBayes using the GTR + G model. The analysis was run for one million generations, with 250,000 generations as burn-in. *Scale bars*: number of substitutions per site. Nodal support is indicated as Bayesian posterior probabilities. Host species are provided in *parentheses*. Outgroup—*M. magellanica* (Opecoelidae)

The Bayesian phylogenetic analysis of 28S rDNA generated a phylogenetic tree with all topologies supported by solid (66–99) posterior probability values (Fig. 2), and all members of *Brachylecithum* were clustered in this tree with 99 % branch support. The tree revealed two well-supported clades. The first included the *Lyperosomum* group with *L. collurionis* from the Eurasian blackcap, and the second contained two subclusters: one with *Dicrocoelium dendriticum* and *D. hospes*, the other with the remaining taxa, i.e., the *Brachylecithum* group. The members of *Brachylecithum* formed two clades: one included *B. grummti* (with weak 54 % branch support) and the other consisted of two clearly separated and well-supported (100 %) clusters, one with *B. capilliformis*, *B. lobatum*, *B. strigis*, and *B. glareoli* and the other formed by *B. kakea* and *B. laniicola* (Fig. 2).

The analysis of the sequences obtained from *Brachylecithum* showed differences within the 28S region of rDNA. The 28S rDNA-based estimate of evolutionary divergence between the species of *Brachylecitum* ranged from 0.001 to 0.077 base substitution differences per site for *B. kakea*/*B. laniicola* and *B. capilliformis*/*B. grummti*, respectively.

**Fig. 3:**
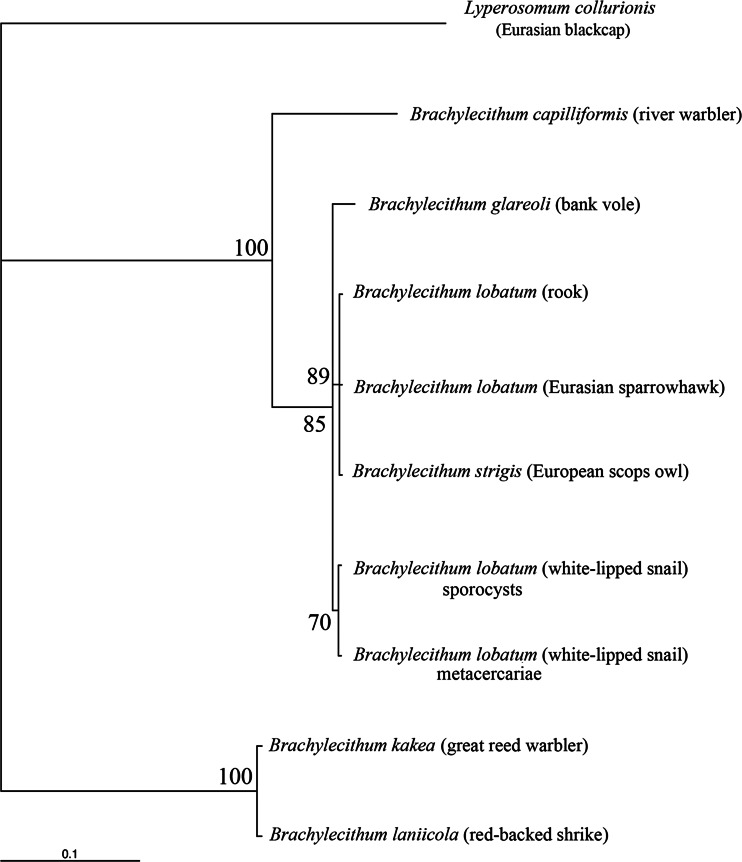
Bayesian analysis of the partial mitochondrial protein-coding gene *cox*1 (data as amino acids) derived from nine isolates of *Brachylecithum* spp. Tree constructed using the HKY + G model. The analysis was run for two million generations; 500,000 generations were discarded as burn-in. The *branch-length scale* indicates the number of substitutions per site. Nodal support is indicated as Bayesian posterior probabilities. Host species are provided in *parentheses*. Outgroup—*Lyperosomum collurionis*

**Fig. 4:**
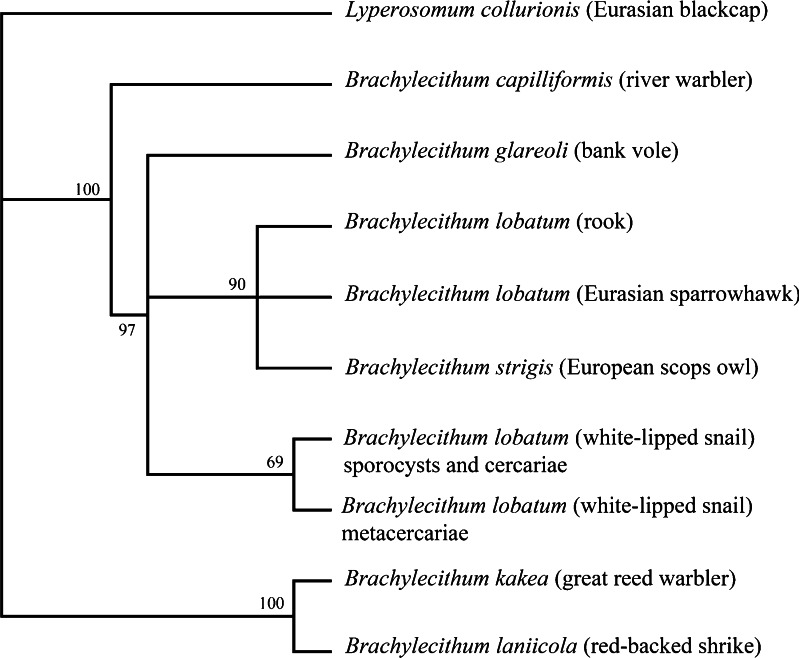
Bayesian analysis of partial sequence 28S rDNA + partial sequence *cox*1 data of nine members of the *Brachylecithum* genus. Tree constructed with MrBayes using the GTR + G model for 28S rDNA and HKY + G for *cox*1. The analysis was run for one million generations, with 250,000 generations as burn-in. *Scale bars*: number of substitutions per site. Nodal support is indicated as Bayesian posterior probabilities. Host species are provided in *parentheses*. Outgroup—*Lyperosomum collurionis*

The *cox*1 sequence analysis yielded a generally similar tree topology, but the isolates of *B. lobatum*/*strigis* from bird hosts clustered in a clade distinct from the rodent-derived *Brachylecithum glareoli* (Fig. 3). The topology of the tree generated by the Bayesian inference in the concatenated dataset confirmed the relationships within *Brachylecithum* observed for the mitochondrial *cox*1 gene (Fig. 4). According to our results, we propose to synonymize *B. strigis* with *B. lobatum*. The genetic differences observed for 28S rDNA and *cox*1 confirm the independent status of *B. capilliformis* and indicate a distinct group within the genus *Brachylecithum*, containing *B. kakea* and *B. laniicola*.

The cercarial and metacercarial isolates from the snails showed 100 % similarity to *B. lobatum* in both genetic markers. This places the larval stages in *B. lobatum*. This is the observation of *Cepaea* snails as intermediate hosts of this trematode.

## Discussion

The phylogenetic analysis presented in this study is still limited to a few representatives of the family Dicrocoeliidae; however, it provides a strong support for the *Dicrocoelium-Brachylecithum* clade and the relationship between *Lyperosomum* spp. and *Dicrocoelium-Brachylecithum* shown in two recent papers (Ribas et al. 2012; Hildebrand et al. 2015).

The phylogenetic tree based on the concatenated 28S rDNA and *cox*1 sequences shows a close relationship between *B. capilliformis* and *B. lobatum*; likewise, *B. kakea* and *B. laniicola* are sister groups. Perhaps, the fact could be explained by their host ecology. In their long-term study, Sitko et al. (2006) recorded *B. kakea* and *B. lanicola* from *Acrocephalus arundinaceus* and *Lanius collurio* only in spring, just after the birds’ arrival (J. Sitko, unpublished data), suggesting that they were non-European species, unable to complete their life cycle in the nesting grounds.

Future molecular studies including more representatives of different genera, for example, *Brachylecithum* and *Lyperosomum*, as well as related species, may explain the relationships within the Dicrocoeliidae and their subfamilies. These analyses would better clarify the phylogenetic position of the dicrocoeliid genera and also the relationships among the species currently included in some of them, i.e., *Brachylecithum*, *Lutztrema*, or *Lyperosomum*, as shown by the results of Hildebrand et al. (2015). According to the previously mentioned and current studies, a similar molecular analysis of non-European *Brachylecithum* species related to the *B. grummti* and *B. kakea*/*B. laniicola* group, and also *Lutztrema* spp., is necessary to understand the relationships within this large genus.

The molecular characteristics of representatives of *Brachylecithum* originating from different hosts show that *B. lobatum* is widely distributed and occurs in birds of the central part of Europe. It parasitizes various hosts, not necessarily closely related to each other, i.e., Corvidae, Accipitridae, or Strigidae. In contrast, the remaining members of *Brachylecithum* analyzed in this study seem to be more host-specific.

The molecular identification of *B. lobatum* in land snails of the genus *Cepaea* is the first indication of the intermediate host for this species. Additionally, it is the first report on all larval stages of *B. lobatum* found in the wild. All the previous studies on life cycles of members of this genus were performed in experimental conditions without obtaining the metacercariae (Carney 1970, 1972, 1974; Denton 1945; Kingston 1965; Timon-David 1957). Only one life cycle of *Brachylecithum* was described completely, i.e., *B. mosquenis*; however, according to the redescription by Sitko and Okulewicz (2002), the species is actually a representative of the genus *Brachydistomum* and uses ants as the second intermediate hosts. That has never been observed in any other *Brachylecithum* species, despite the attempts (Carey 1972). The simultaneous presence of sporocysts with well-developed cercariae and metacercariae (encysted inside the sporocyst) in the same snail specimen (Fig. 1) is the first case of life cycle abbreviation in the Dicrocoeliidae (Poulin and Cribb 2002). According to Poulin and Cribb (2002) and Galaktionov and Dobrovolskij (2003), there are a few known types of truncations of life cycles among trematodes, including the abovementioned. The authors proposed “the rare or missing host hypothesis” to explain this shorter cycle by seasonal migrations or fluctuations in host abundance.

**Fig. 5:**
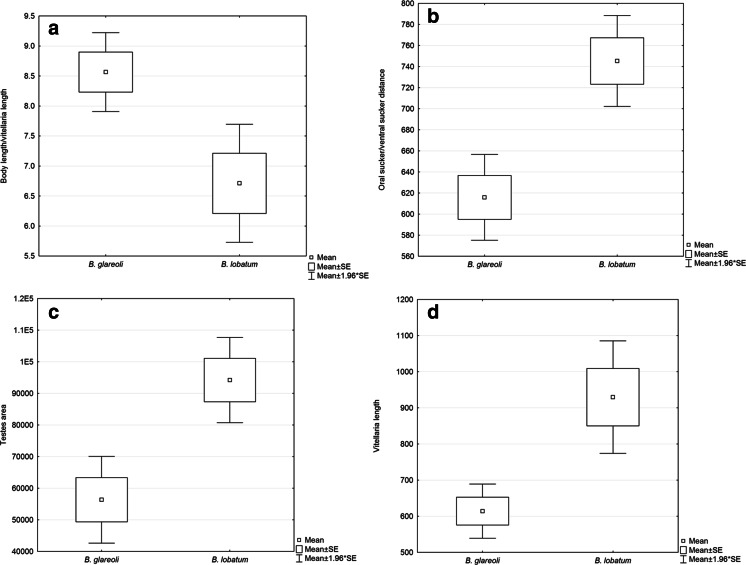
Comparison of morphometric characters of *B. glareoli* and *B. lobatum*. **a** Ratio of body length to vitellaria length, **b** distance between oral and ventral suckers, **c** testis area, and **d** vitellaria length

*B. glareoli*, a dicrocoeliid parasitizing small mammals, was described as a distinct species based on morphological discriminant analysis of all known dicrocoeliids recorded from rodents or insectivores (Hildebrand at al. 2007); the same is true of a few *Brachylecithum* species which occur in Holarctic birds and show partial similarity in such morphological characters as position and shape of gonads or shape and proportion of suckers. However, in our phylogenetic analyses, *B. glareoli* showed a high level of similarity with *B. lobatum*, with five nucleotides differing among the *cox*1 sequences, compared to *B. glareoli* and isolates of *B. lobatum* from different (definitive and intermediate) hosts. Additionally, we analyzed the morphological traits of the two species. Overall, 20 individuals of both species were measured as described by Hildebrand et al. (2007). The mean values were analyzed using *t* test (Statistica 10). The two species differ significantly in the most important taxonomic features. *B. lobatum* has vitellaria 30 % longer than *B. glareoli* (*p* < 0.01), almost 50 % larger testes (*p* < 0.01), and 20 % longer distance between oral and ventral suckers (*p* < 0.001) (Fig. 5). Considering both statistical and molecular data, we recognize *B. glareoli* as a still valid taxon.

**Fig. 6:**
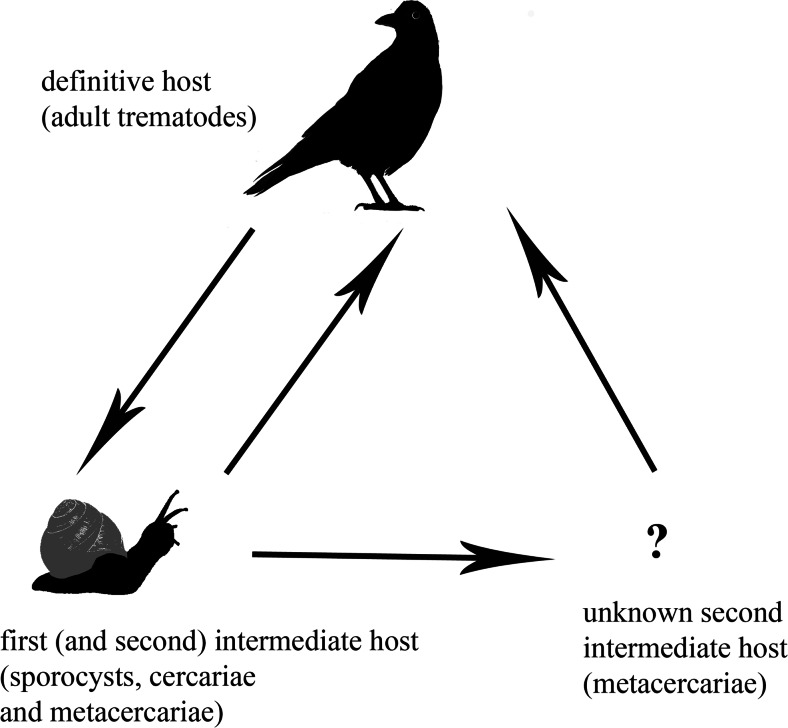
Possibilities of the *B. lobatum* life cycle

The reported molecular similarity between B*. lobatum* and *B. glareoli* may suggest that the *B. glareoli*-bank vole association is probably quite a new host-parasite relationship. This new association is probably derived from the well-established and common system of *B. lobatum*-bird host, as a result of host switching. In this study, we presented the evidence that *B. lobatum* could abbreviate its life cycle in some environmental conditions and probably could complete the life cycle in a way which is typical of the vast majority of digeneans, i.e., involving three hosts (Fig. 6). It seems more likely that the shortening of the life cycle of *B. lobatum*, which was recorded in the present study, was one of the factors which facilitated the switch of the fluke between bird and rodent hosts.
